# The relationship between social class and subjective well-being: A serial mediation model

**DOI:** 10.3389/fpsyg.2022.1002585

**Published:** 2022-10-10

**Authors:** Youjuan Hong, Xuemei Wang, Liting Liu, Yankui Su, Lijun Chen, Rong Lian, Meiling Liao

**Affiliations:** ^1^School of Nursing, Fujian Medical University, Fuzhou, China; ^2^School of Preschool Education, Fujian Preschool Education College, Fuzhou, China; ^3^School of Management Studies, Shanghai University of Engineering Sciences, Shanghai, China; ^4^College of Foreign Languages, Fujian Normal University, Fuzhou, China; ^5^School of Humanities and Social Sciences, Fuzhou University, Fuzhou, China; ^6^School of Psychology, Fujian Normal University, Fuzhou, China; ^7^The School of Health, Fujian Medical University, Fuzhou, China

**Keywords:** social class, victim justice sensitivity, envy, subjective well-being, serial mediation model

## Abstract

Despite recent research associating social class with subjective well-being (SWB), the relationship between the two, manifested through victim justice sensitivity and envy, has not been properly investigated. Guided by social comparison and social cognitive tendency theories, we explored the direct and indirect relationships between social class and SWB among Chinese undergraduate students. This study employed a cross-sectional, questionnaire-based research design. 1,405 undergraduate students completed questionnaires regarding subjective social class, victim justice sensitivity, envy, and SWB. The results showed that social class was positively related to SWB and negatively associated with victim justice sensitivity and envy. Victim justice sensitivity was negatively related to SWB, victim justice sensitivity was positively related to envy, and envy was negatively associated with SWB. Social class correlated with SWB through three paths: the mediating role of victim justice sensitivity, the mediating role of envy, and the serial mediating roles of victim justice sensitivity and envy. The results indicate that social class could contribute to college students’ SWB through the mechanisms of victim justice sensitivity and envy. This study advances the understanding of how the relationship between social class and college students’ SWB operates. Furthermore, the findings will facilitate the promotion of college students’ SWB.

## Introduction

Pursuing subjective well-being (SWB) has historically been one of the strongest motivators of human behavior (Akdogan and Cimsir, 2019). Many scientists have argued that health, income, self-esteem, perceived social justice, and optimism correlate with SWB (Diener et al., 2012; Yang and Penghui, 2018). Happiness is one of humankind’s most advanced emotional experiences and is highly related to the social class to which the individual belongs (Howell and Howell, 2008). Decades of research have shown that social class positively correlates with SWB; individuals of higher social classes experience substantially greater SWB (Yu and Blader, 2019). Why does social class affect SWB? Why do lower social classes experience less SWB? Prior research has not extensively considered this issue and instead has focused on the magnitude of social class’ relationship with SWB. The current research investigates the mechanisms by which social class correlates with SWB.

Previous research found that social class negatively correlates with victim justice sensitivity (Hong et al., 2020). Specifically, lower social classes are associated with enhanced victim justice sensitivity. This is because the relative lack of resources makes individuals in lower classes more vulnerable than those in upper classes are, with greater reactivity to threats, such as discrimination (Varnum and Kitayama, 2017). Victim justice sensitivity was a vulnerability and a stress factor that may add to developing anxiety (Bondü and Inerle, 2020). In addition, envy has also been identified as a cause of unhappiness and an obstacle to SWB (Carlin and Capps, 2012). Therefore, victim justice sensitivity and envy may play vital roles in the relationship between social class and SWB. Moreover, victim justice sensitivity and envy are correlated (Gollwitzer et al., 2005; Hong et al., 2021). It is reasonable to assume that social class, victim justice sensitivity, envy, and SWB are all correlated with one another. For instance, being of a lower social class may increase victim justice sensitivity, and victim justice sensitivity may increase envy, decreasing happiness. Previous studies have only explored the relationship between social class and victims’ justice sensitivity and jealousy. However, it is unclear how these variables relate to college students’ SWB.

Therefore, in the current study, we propose a model for the parallel and serial mediating effects of victim justice sensitivity and envy on the association between social class and SWB. A more detailed description of the reasoning supporting the current study and a further explanation regarding the study variables are included below.

## Background

### Social class and subjective well-being

Social class refers to an individual’s material resources and perceived rank within the social hierarchy (Kraus et al., 2009). Researchers often assess the construct as objective social class (OSC) or subjective social class (SSC); OSC is measured by income, education, and profession (Goodman et al., 2001), while SSC refers to the individual’s perceived rank relative to others in society (Kraus et al., 2009). Several studies have demonstrated that SSC is a relatively better predictor of psychological outcomes than OSC (Kraus et al., 2009). Therefore, in the present study, we subjectively assessed social class and primarily focused on mechanisms of the relationship between SSC and SWB.

Social class is manifested in a wide range of signals, such as wealth and education. For instance, higher social classes enjoy many advantages, including greater wealth, better employment opportunities, occupational prestige, and improved education (Kraus et al., 2009). Conversely, lower social classes possess fewer resources and less wealth and opportunity (Kraus et al., 2011a). As a result, higher social classes are given greater status, regarded as more socially attractive, and command more respect (Henrich and Gil-White, 2001).

Previous studies have consistently found a positive relationship between social class and SWB (Yu and Blader, 2019; Evans and Rubin, 2022). People of lower classes experience more stress than those of higher classes (Sidorchuk et al., 2016). They suffer from more psychological symptoms (such as depression) and negative emotions (Adler and Rehkopf, 2008). For example, a 13-year longitudinal follow-up study found that depressive patients in lower social classes were more likely to worsen (Bromberger et al., 2017). Chen and Matthews (2001) found that children and adolescents from lower social classes experienced more hostility and anger in ambiguous situations. Being of a higher social class enhances SWB because it contributes to fulfilling psychological needs that are key antecedents of happiness, such as respect, autonomy, mastery, etc (Diener et al., 2010), and being of a lower social class is related to reduced SWB (Anderson et al., 2012). Therefore, based on this evidence, we propose the following hypothesis:

*Hypothesis 1:* Social class is positively associated with SWB.

### The mediating roles of victim justice sensitivity and envy

Victim sensitivity refers to an individual’s tolerance of unjust treatment toward themselves (Schmitt et al., 2005, 2010). Social class may predict victim justice sensitivity. The theory of social cognitive tendency assumes that the social class context elicits reliable social cognitive patterns (Kraus et al., 2012). Lower-class individuals are characterized by a contextual, externally-oriented cognitive relationship with the world, while upper-class individuals are characterized by a solipsistic, individualistic cognitive association (Kraus et al., 2012). Lower-class individuals with contextualist tendencies focus on external, uncontrollable social forces and other individuals who influence their life outcomes (Kraus et al., 2012). They pay more attention to whether society is just and fair. In contrast, upper-class individuals with solipsistic social cognitive tendencies focus on their internal state, goals, motivations, and emotions. They pay less attention to information related to social injustice and are less sensitive to its presence.

In addition, previous studies have shown that victim justice sensitivity may make individuals more sensitive to and vigilant about injustice (Bondü and Esser, 2015; Mario et al., 2015). Lower social classes have been linked to greater reactivity to threats because of their comparative lack of resources (Varnum and Kitayama, 2017). Thus, such individuals are always at a disadvantage in competitive situations; therefore, a sense of unjustness is easily elicited. For example, Hajat et al. (2010) found that lower-class individuals must always be prepared to respond to threats in life compared to higher social classes. That is, lower social classes are more sensitive to threats (Hajat et al., 2010). Wu (2017) examined the relationship between social class and victim justice sensitivity using an initiation task in which participants played the roles of lower-or higher-class individuals. They found that victim sensitivity was significantly lower in the higher rather than lower social classes. Lower-class individuals were concerned about being treated unfairly, resulting in a pronounced motivation for self-protection (Gerlach et al., 2012). Therefore, it is reasonable to believe that social class is negatively associated with victim justice sensitivity.

Victim sensitivity also correlates with SWB. First, highly victim-sensitive individuals can easily become disturbed in terms of their emotional well-being and cognition when faced with injustice (Schmitt et al., 2005; Kals and Maes, 2013). Individuals with low victim sensitivity are relatively unaffected by justice-related matters. Feelings of distress directly decrease one’s happiness. For example, people who score high on victim sensitivity worry that their investments may be exploited by others (Rothmund et al., 2011). Long periods of worry hurt happiness. Therefore, it is reasonable to assume that victim justice sensitivity may mediate the relationship between social class and SWB. Indeed, the mediation effect of victim justice sensitivity operating between social class and SWB is supported by empirical evidence. Guo et al. (2015) examined the relationships connecting social class, victim justice sensitivity, and social justice, finding that victim justice sensitivity mediated the relationship between social class and social justice, and social justice correlated with SWB. People who felt that society was unfair experienced less happiness in the study (Tian et al., 2017). Thus, the following hypothesis is proposed:

*Hypothesis 2:* Victim justice sensitivity mediates the relationship between social class and SWB.

Social class may have a negative relationship with envy. Envy is an emotion characterized by the unpleasant experience of hostility, inferiority, and resentment toward those who possess something desirable (Smith and Kim, 2007). Higher-class individuals have more material resources and a higher social rank than do the lower classes, with fewer resources and a more subordinate rank in society. Therefore, individuals of lower social classes have a stronger sense of inferiority and deprivation than those of higher social classes. A sense of deprivation can quickly produce envy (Smith, 1991). Moreover, lower-class individuals have less personal control and are more sensitive to external threats; thus, if they are aware of their disadvantage, they are more likely to experience envy (Vecchio, 2000; Piff and Robinson, 2017; Chen et al., 2021).

The experience of envy is closely associated with SWB (Ding et al., 2017). Envy can trigger SWB along two paths: feeling displeased or a cognitive emotion obtained through self-evaluation (Lim and Yang, 2019). On the one hand, many psychologists have argued that envy is an unpleasant feeling experienced when one realizes that others have something they are trying to gain but currently lack (Ortony et al., 1988; Parrott and Smith, 1993; Smith and Kim, 2007). Envy can also embed a sense of injustice in response to either an unfair advantage received by another or a disadvantage experienced by oneself (Ganegoda and Bordia, 2018). On the other hand, upward social comparisons are often the foundation of envy (Reh et al., 2017). When envy is triggered, the individual recognizes that they are being rated as less than the comparison target, causing them to feel depressed (Lim and Yang, 2019). Consequently, envy directly decreases individuals’ SWB (Ding et al., 2017). Therefore, it is reasonable to assume that envy may mediate the relationship between social class and SWB. Thus, we propose:

*Hypothesis 3:* Envy mediates the relationship between social class and SWB.

Both victim justice sensitivity and envy are associated with SWB. In addition, studies have found that victim justice sensitivity and envy are related. This suggests that victim justice sensitivity and envy may influence one another and decrease SWB. If so, two potential mediate effects should be considered. One possibility is that victim justice sensitivity decreases SWB through envy. The other is that envy influences victim justice sensitivity, decreasing SWB. The former seems plausible because it has been found that individuals with high victim sensitivity believe that society is unfair (Bondü and Esser, 2015; Mario et al., 2015). What’s more, a sense of injustice is a predictor of envy (Feather and Sherman, 2002). In a study examining the role of envy in the relationship between a sense of justice and SWB, Li (2012) found that a sense of injustice is negatively correlated with envy. Envy was also found to mediate the relationship between a sense of injustice and SWB. These findings suggest that it is more reasonable to treat victim justice sensitivity as a factor affecting envy than to do the opposite. Lower social classes experience a sense of injustice (Gerlach et al., 2012). Therefore, it is likely that victim justice sensitivity and envy mediate the linkage between social class and SWB. Specifically, social class may be a predictor of victim justice sensitivity, and victim justice sensitivity may influence envy and decrease SWB. Thus, we propose:

*Hypothesis 4:* Victim justice sensitivity and envy play serial mediating roles in the relationship between social class and SWB.

### The present study

**Figure 1:**
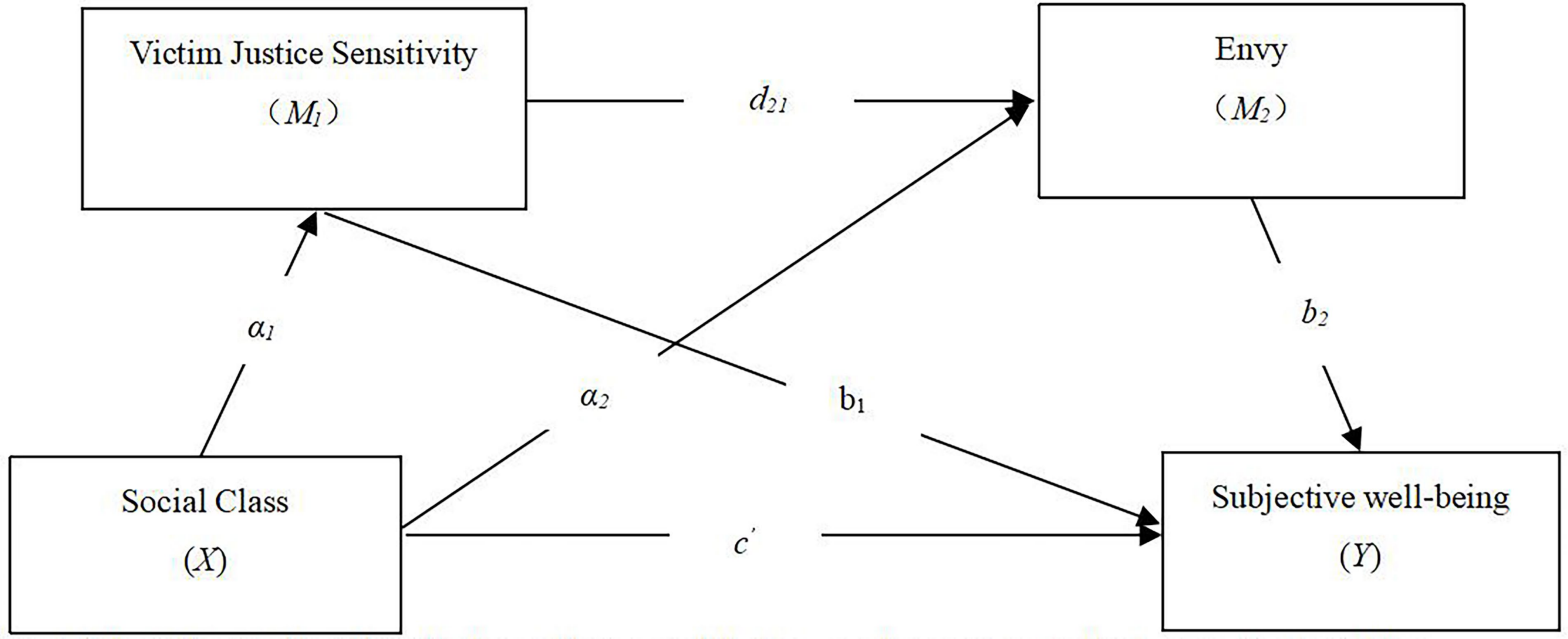
A hypothesized serial multiple mediation model. Age and gender were considered as control variables. *p < 0.05, **p < 0.01, ***p < 0.001.

Previous research has shown that social class is a predictor of SWB. Victim justice sensitivity was found to mediate the relationship between social class and social justice, social justice was determined to correlate with SWB (Guo et al., 2015). Meanwhile, victim justice sensitivity was correlated with envy. Exploring the mediating roles of victim justice sensitivity and envy will help establish a better understanding of the relationship between social class and SWB. However, there has been no research investigating the linkage of social class and SWB through victim justice sensitivity or envy. Theoretical propositions and previous research have indicated that social class may correlate with SWB through victim justice sensitivity and envy. Therefore, the present study investigated the association between social class and SWB and explored whether victim justice sensitivity and envy mediate this possible relationship in a causally-connected way. The results will contribute to the current literature by extending our understanding of the mechanism that connects social class and SWB (see Figure 1).

## Materials and methods

### Participants and procedure

According to (Roscoe et al., 2018) a general rule of thumb is that a sample size between 30 and 500 participants is appropriate and sufficient for conducting a research survey. Therefore, we recruited 400 to 500 subjects at each university. 1,405 undergraduate students (66.6% female) were recruited from Fujian Normal universities, Fujian Medical Universities, Fujian Agriculture and Forestry Universities in the southern city of China. These college students come from different professional fields, including teacher training, medicine, physics, agriculture and so on.

Their mean age was 19.49 (*SD* = 1.18), ranging from 18 to 21 years. Of the total, 354 (25.1%) were first-year students, 360 (25.6%) were in their second year, 370 (26.33%) were in their third, and 321 (22.8%) were in their fourth. This study employed a cross-sectional, questionnaire-based research design. Due to the time, cost, and accessibility factors, the convenience sampling method is used as this method provides the highest response level while saving resources and timely feedback (Etikan et al., 2016).

The investigation was conducted from March 01, 2021 to July 30, 2021. Here, trained researchers administered the self-report questionnaires in the classroom or library, after obtaining informed consent and emphasizing the anonymity of responses. Participants answered all the measurements in Chinese. Permission to implement the study was granted by the Academic Ethics Committee of Fujian Medical University. After surveys were completed, participants received a token gift of small monetary value.

### Measures

#### Subjective social class scale

MacArthur Scale of subjective social status (Adler et al., 2000) was used to measure subjective social class. It is one of the most ubiquitous instruments deployed in the literature. The scale consists of drawing a ladder with 10 rungs, each given a number between 1 and 10, representing different income levels, education, and occupational status. At step 10 are people who are the best off – those who have the most money, the most education, and the most respected jobs. At step 1 are the people who are worst off – those who have the least money, least education, and the least respected jobs or no job. Participants are instructed to select the number representing their perception of their family’s placement on this 10-point social scale. This measure is widely used and has demonstrated adequate test–retest reliability, and the test–retest reliability was 0.62 (Operario et al., 2004; Tan et al., 2021).

#### Victim justice sensitivity scale

The Victim Justice Sensitivity Inventory (Schmitt et al., 2010) consists of 10 items, with answers given on a scale ranging from 1 (“totally disagree”) to 6 (“totally agree”). Higher scores represent stronger victim justice sensitivity. An example item is as follows: “I get upset when someone else gets what should have been mine.” This scale demonstrated high reliability and validity in a sample of Chinese college students (Hu et al., 2020). The results of confirmatory factor analysis are as follows: χ^2^/df = 2.44, RMSEA = 0.05, CFI = 0.94, NFI = 0.90, GFI = 0.96, indicating that a one-factor model fitted reasonably well. The scale was found to have a high reliability level in the current study (Cronbach’s alpha = 0.84).

#### Envy scale

The Envy Scale (Smith and Kim, 2007) consists of eight items, with answers given on a scale ranging from 1 (“totally disagree”) to 5 (“totally agree”). Examples include: “It is so frustrating to see some people always having a good time” and “Many of my friends have a better life than me.” This scale demonstrated high reliability and validity in a sample of Chinese college students (Ding et al., 2017). The results of confirmatory factor analysis are as follows: χ^2^/df = 5.14, RMSEA = 0.06, CFI = 0.94, NFI = 0.93, GFI = 0.97, indicating that a one-factor model fitted reasonably well. The scale was found to have a high reliability level in the current study (Cronbach’s alpha = 0.71).

#### Subjective well-being scale

The positive and negative affect schedule (PANAS; Watson et al., 1988) assesses the affective dimension of SWB. It consists of positive affect (PA) and negative affect (NA) sub-scales. The PA scale consists of 10 items, with answers given on a scale ranging from 1 (“very slightly or not at all”) to 6 (“extremely”). An example item is “interested.” The NA scale also includes 10 items, with an example being “upset.” Higher scores reflect a greater positive or negative effect. The Satisfaction with Life Scale (SWLS; Diener et al., 1985) assesses the cognitive dimension of SWB. The measure consists of five items, with answers given on a scale ranging from 1 (“strongly disagree”) to 6 (“strongly agree”). Higher scores indicate greater satisfaction with life. An example item is: “In most ways, my life is close to my ideal.” Finally, the score of the SWB was computed by adding the standardized SWLS and PA scores and then deducting the standardized NA score (Sheldon and Elliot, 1999). In the current study, the PA, NA, and SWLS scales demonstrated high reliability (Cronbach’s alpha = 0.91, 0.90, and 0.84, respectively). We conducted a CFA on a 3-factor model (life satisfaction, positive affect, and negative affect).The results of CFA are as follows: χ^2^/df = 5.15, RMSEA = 0.07, CFI = 0.93, NFI = 0.91, GFI = 0.89, and TLI = 0.92, indicating that factors model fitted reasonably well.

## Results

### Preliminary analysis

The data’s descriptive statistics and main analyses (i.e., regression analysis and serial multiple mediation) were conducted using the IBM SPSS-21. Harman’s single-factor test was used to examine the effect of common method bias. The results showed nine factors with an eigenvalue greater than 1; the interpretation rate for the first factor was 23.13% (Podsakoff et al., 2003). Thus, no common method variance was found in the current study.

The Pearson correlations and means and standard deviations of all study variables are presented in Table 1. Social class was positively correlated with SWB and negatively correlated with victim justice sensitivity and envy, victim justice sensitivity was negatively correlated with SWB and positively correlated with envy, and envy was negatively correlated with SWB.

**Table 1:** Means, standard deviations, and correlations for all study variables (*N* = 1,405).

Variable	Range	*M*	*SD*	1	2	3	4	5	6
1.Subjective social class	1–10	4.74	1.38	1					
2.Victim justice sensitivity	1–6	4.23	0.75	−0.15^***^	1				
3.Envy	1–5	2.90	0.57	−0.22^***^	0.47^***^	1			
4.Subjective well-being	1–6	4.01	1.82	0.23^***^	−0.40^***^	−0.55^***^	1		
5.Age		19.49	1.11	0.01	0.06^*^	0.02	−0.01	1	
6.Gender		–	–	0.05	0.11^*^	0.03	−0.05	0.02	1

Gender: 0 = Male; 1 = Female. ^*^p < 0.05, ^**^p < 0.01, ^***^p < 0.001.

### Hypothesis testing

The prediction of SWB *via* the variables of social class, victim justice sensitivity, envy, age, and gender (coded as 0 = female, 1 = male) was conducted by multiple regression analysis. The results are provided in Table 2. The direct relationships between social class and victim justice sensitivity, and social class and envy were tested in Models 1 and 2 (see Table 2). The regression results based on victim justice sensitivity as an output variable (see Model 1) showed that a high social class was associated with a low level of victim justice sensitivity (β = −0.14, *p* < 0.001). As expected, in Model 2, with envy as the dependent variable, social class negatively influenced envy (β = −0.16, *p* < 0.001).

**Table 2:** Model results.

Variables		Model 1				Model 2				Model 3	
	VJS		Envy		SWB
	β	*SE*	*t*		β	*SE*	*t*		β	*SE*	*t*
Constant	−0.91	0.42	−2.17		0.08	0.40	0.20		−0.18	0.39	−0.46
Gender	0.08	0.05	1.57		0.05	0.05	1.01		−0.03	0.04	−0.71
Age	0.05	0.02	2.46		−0.01	0.02	−0.13		0.01	0.02	0.26
SSC	−0.14	0.02	−5.68^***^		−0.16	0.02	−6.70^***^		0.11	0.02	4.67^***^
VJS					0.49	0.03	19.00^***^		−0.20	0.03	−7.10^***^
Envy									−0.46	0.03	−17.91^***^
*R* ^2^		0.03				0.24				0.34	
*F*		30.00^***^				114.01^***^				145.09^***^	

*N* = 1,405.SSC, subjective social class; VJS, victim justice sensitivity; SWB, subjective well-being. *p < 0.05, **p < 0.01, ***p < 0.001.

Table 2 shows that the variables for social class, victim justice sensitivity, envy, age, and gender predicted SWB at a level of 34% [*R^2^* = 0.34, *F*(5,1,399) = 145.09, *p* < 0.001]. Social class, victim justice sensitivity, and envy appeared to be significant predictors of SWB, while age and gender were not.

The relationship between social class and SWB *via* victim justice sensitivity and envy was determined using the SPSS PROCESS v2.16.3 macro (see Model 6) developed by Hayes (2013). A 95% bias-corrected confidence interval (CI) based on 5,000 bootstrap samples was used to examine the mediating effects, considered statistically significant if the CI did not include zero. The results are shown in Table 3.

**Table 3:** Effects and 95% confidence intervals for models.

	Effect	Bootstrap SE	Boot LLCI	Boot ULCI
Total effect	0.24			
Direct effect	0.11	0.02	0.06	0.15
Total indirect effect	0.13	0.02	0.09	0.17
Indirect effect 1	0.03	0.01	0.02	0.05
Indirect effect 2	0.03	0.01	0.02	0.05
Indirect effect 3	0.07	0.01	0.05	0.10

The path of indirect effect 1 is social class → victim justice sensitivity → SWB; the path of indirect effect 2 is social class → victim justice sensitivity → envy → SWB; and the path of indirect effect 3 is social class → envy → SWB.

**Figure 2:**
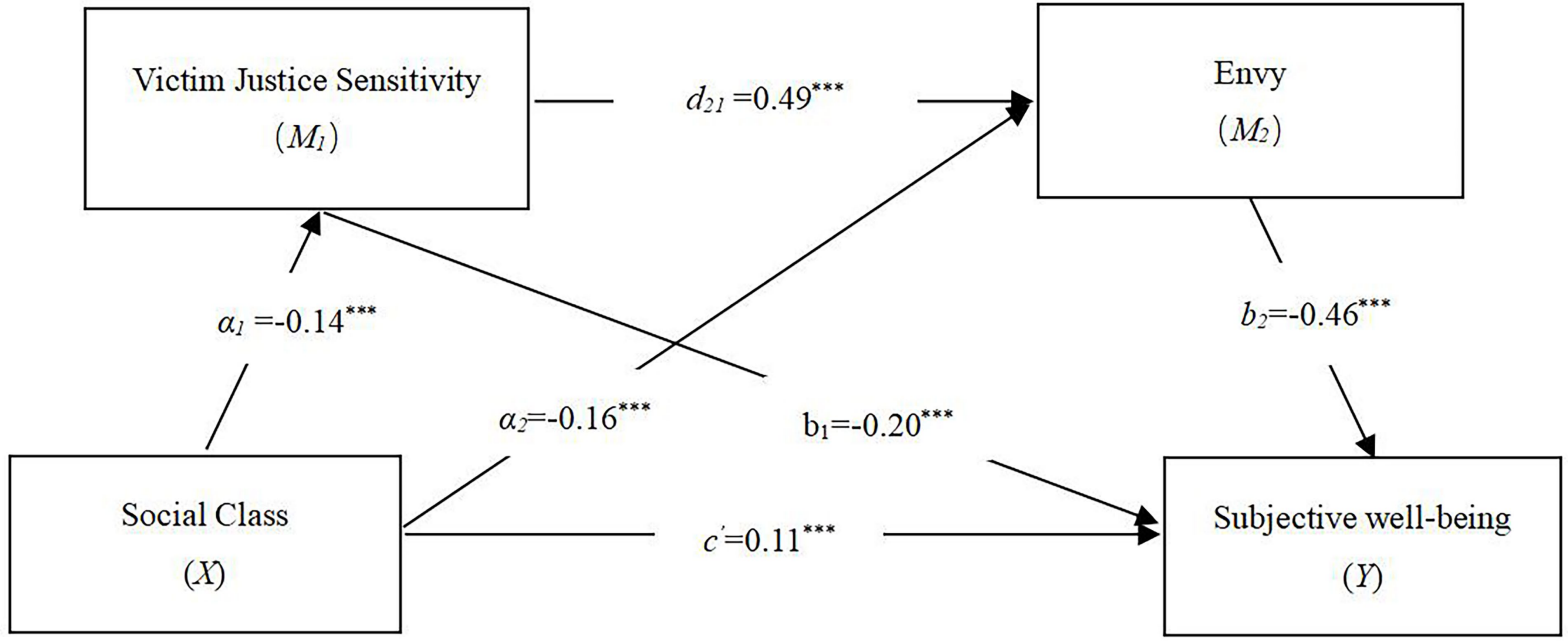
Path analysis models of the effects of social class, victim justice sensitivity, envy, and subjective well-being in Chinese undergraduates. ***p < 0.001.

The statistical diagram in Figure 2 depicts this serial mediation model in which social class (*X*) was found to correlate with SWB (*Y*) along four pathways (i.e., a_1_b_1_, a_2_b_2_, a_1_d_21_b_2_, and c′). The arrows in the figure display the paths of the tested model, and a_1_, a_2_, b_1_, b_2_, d_21_, and c′ indicate the path coefficients.

First, Table 3 shows that the relationship between social class and SWB was significant (c′, = 0.11, *p* < 0.001). Second, the relationship between social class and SWB was significant through victim justice sensitivity (i.e., *X → M_1_ → Y*, a_1_b_1_ = 0.03). The mediating effect of envy operating between social class and SWB was significant (i.e., *X → M_2_ → Y*, a_2_b_2_ = 0.07). Last, the serial mediation effect of victim justice sensitivity and envy was also significant (i.e., *X → M_1_ → M_2_ → Y*, a_1_d_21_b_2_ = 0.03). Both victim justice sensitivity and envy were found to mediate the association between social class and SWB, supporting Hypotheses 1, 2, 3, and 4.

In these analyses, we observed victim justice sensitivity and envy mediating effects on the relationship between social class and SWB. Table 3 shows the direct effect of the linkage between social class and SWB was 0.11. The total mediating effect of victim justice sensitivity and envy was 0.13. Specifically, the mediating effect consisted of three pathways. (1) The mediating effect of victim justice sensitivity was 0.03 (95% CI: 0.02–0.5). (2) The mediating effect of victim justice sensitivity and envy was 0.03 (95% CI, 0.02–0.05). (3) The series of mediating effects from envy was 0.07 (95% CI, 0.05–010). All mediating effect paths were significant. Furthermore, the mediating effect path of “social class → envy → SWB” was bigger than the mediating effect of “social class → victim justice sensitivity→ SWB” (β = 0.04, *p* < 0.001), or the mediating effect of “social class → victim justice sensitivity→ envy→ SWB” (β = 0.03, *p* < 0.001). The largest mediating effect path was “social class → envy → SWB.” By comparing the three mediating paths results, it suggests that lower-class individuals are more likely to suffer from a decreased SWB, primarily due to increased envy (see Table 3).

## Discussion

Based on social comparison theory and the theory of social cognitive tendency, the present study investigated the relationship between social class and SWB and determined whether victim justice sensitivity and envy acted as mediators in that relationship. The results show there was a significant association between SSC and SWB. What’s more, the evidence supports the three hypothesized mediating effects: (1) “social class → victim justice sensitivity → SWB,” (2) “social class → envy → SWB,” and (3) “social class → victim justice sensitivity → envy → SWB”.

This study found that social class has a significantly positive correlation with SWB. The result follows previous research (Sidorchuk et al., 2016). Compared to those of higher social classes, lower-class individuals suffer from a wide range of disadvantages, are less likely to have access to economic resources (Kraus et al., 2011b), and often experience a sense of low status. They are also less likely to meet specific fundamental psychological needs, such as relatedness, autonomy, and mastery (Diener et al., 2010). Therefore, they experience substantially reduced SWB.

This study found that both victim justice sensitivity and envy mediated the relationship between social class and SWB. These findings support social comparison theory and the theory of social cognitive tendency. The results imply that victim justice sensitivity and envy play a significant role in the relationship between social class and SWB. The significance of the mediating effect of victim justice sensitivity suggests that being of lower social class results in significantly less happiness by increasing the individual’s victim justice sensitivity. Lower-class individuals with contextual cognitive patterns are more likely to believe in social injustice and oppose a current social situation they perceive as unfair (Kraus et al., 2012). Therefore, lower-class individuals are more likely to become sensitive to victim justice, and such individuals worry about perceived losses and experience low SWB (Rothmund et al., 2011). This finding is essential for revealing the significant role of victim justice sensitivity in the level of unhappiness of individuals of lower social classes. Previous studies have found the correlation between social class and victim justice sensitivity, and the current study further found the relationship between them and SWB. The current study’s findings provide a new perspective for understanding the relationship between social class and SWB.

The significance of the mediating effect of envy also suggests that being of a lower social class reduces happiness by increasing an individual’s amount of envy. Lower-class individuals are likely to compare themselves to others who are better off, thus leading to such envy. This finding reveals that envy is correlated with being of a lower social class and feeling unhappy about it. Envy is an affective experience consisting of two principal components, one related to feelings of inadequacy and the other to feelings of ill will (Salovey and Rothman, 1991). Lower social classes are more likely to experience hostility and inferiority because of reduced resources, decreasing their happiness.

The serial mediation analysis results support our hypothesis that the relationship between social class and SWB occurs through a process in which lower-class individuals have an increased tendency to be more victim-sensitive, resulting in increased envy and translating into a decreased level of happiness. This result follows studies highlighting that a sense of injustice is an important factor in an individual’s experience of envy and level of SWB (Schaubroeck and Lam, 2004). In contrast to previous research, this study reveals that social class can be associated with SWB through multiple pathways. The results of the current study guide on how to intervene in the SWB of low social class college students (e.g., poor students).

This study benefits the literature on the relationship between social class and SWB. At the same time, it also refers to subsequent studies on the formation mechanism of SWB. This study theoretically reveals the influence of social class on SWB and its internal mechanism, which helps expand our understanding of the factors causing low SWB and broadens the research on SWB of undergraduates. The results also have practical implications. Educators need to understand the information about the subjective social class of college students and pay special attention to the students with low subjective social class (e.g., poor students). Educators and practitioners should focus on the negative effects of victim justice sensitivity and envy in those of lower social classes and take effective measures to reduce their sense of injustice. For instance, in some activities, teachers should try to be fair in distribution, procedure or interaction. Emotion management courses and group counseling activities on the topic can be carried out by reducing individual victim justice sensitivity and envy to enhance the general positive emotional quality of life.

## Limitations and future research directions

This study has several limitations. First, the participants were recruited by convenience sampling, potentially leading to significant selective bias. A randomized sampling study should be conducted in the future. Second, this study relies on self-report measures, and the results were produced based on correlational evidence of observed variables. Thus, the causal relationships among social class, victim justice sensitivity, envy, and SWB cannot be guaranteed. Those who feel unhappy about their life situation (i.e., suffer from a low SWB) may be more inclined to see society as unfair and feel envious of those doing better. To investigate the assumed causal relationships in our serial mediator model, longitudinal studies are needed to explore the long-term relationship between social class, SWB, victim justice sensitivity, and envy. Using an objective measure of social class would also help to rule out the possibility that the current study reflects causal results. Third, in the present study, young undergraduates constituted the majority of the sample, decreasing the generalizability of the findings to a more representative population. Therefore, a broader sample that would represent the Chinese population should be investigated. In addition, the current study only focused on the mediators of victim justice sensitivity and envy. Other undiscovered variables could explain why being of a lower social class may make an individual less happy in the moment. For example, social class has been found to correlate positively with power (Fiske, 2010; Belmi and Laurin, 2016). Finally, it is important to determine if perpetrator and observer justice sensitivity are the same as the effect of victim justice sensitivity on the relationship between social class and SWB. Indeed, future research should consider more factors to provide a more comprehensive picture of the social class-SWB connection.

## Conclusion

The research findings suggest that social class significantly correlates with SWB. In addition, victim justice sensitivity and envy act as serial mediators between social class and SWB, indicating that lower social classes tend to experience more victim justice sensitivity, creating a greater likelihood of envy and resulting in less SWB. Moreover, the research findings show that an individual’s victim justice sensitivity and envy can emerge as a product of being of a lower social class, and such a situation is closely related to the individual’s SWB.

## Data availability statement

The original contributions presented in the study are included in the article/supplementary material, further inquiries can be directed to the corresponding author.

## Ethics statement

The studies involving human participants were reviewed and approved by Academic Ethics Committee of Fujian Medical University. The participants provided their written informed consent to participate in this study.

## Author contributions

YH: conception and design, acquisition of data, and analysis and interpretation of data. XW, LL, YS, and LC: drafting the manuscript or revising it critically for important intellectual content. RL and ML: investigated and resolved. All authors contributed to the article and approved the submitted version.

## Funding

This work was supported by the Fujian Social Science Planning Project under Grant (FJ2018C093).

## Conflict of interest

The authors declare that the research was conducted in the absence of any commercial or financial relationships that could be construed as a potential conflict of interest.

## Publisher’s note

All claims expressed in this article are solely those of the authors and do not necessarily represent those of their affiliated organizations, or those of the publisher, the editors and the reviewers. Any product that may be evaluated in this article, or claim that may be made by its manufacturer, is not guaranteed or endorsed by the publisher.
